# 2-(4-Hydroxy­phen­yl)acetic acid–4,4′-bipyridine (1/1)

**DOI:** 10.1107/S1600536810018842

**Published:** 2010-05-26

**Authors:** Jian-Feng Liu, Guo-Liang Zhao

**Affiliations:** a College of Chemistry and Life Sciences, Zhejiang Normal University, Jinhua 321004, Zhejiang, People’s Republic of China

## Abstract

In the acid mol­ecule of the title complex, C_10_H_8_N_2_·C_8_H_8_O_3_, the acetyl C—C—C—O torsion angle is −32.1 (3)°, and in the mol­ecule of the base, the dihedral angle between the two pyridine rings is 23.41 (10)°. In the crystal structure, inter­molecular O—H⋯N hydrogen bonds link the acid and the base mol­ecules into a one-dimensional triple-helix framework extended along the *b* axis.

## Related literature

For related functional complexes, see: Han *et al.* (2009 ▶). For hydrogen-bond motif structures, see: Tomura & Yamashita (2001 ▶). 
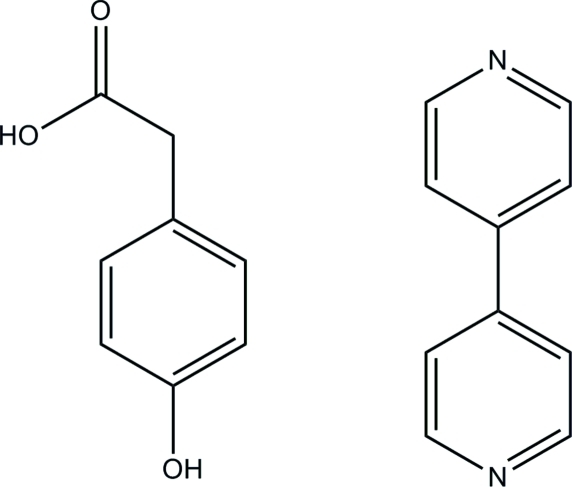


## Experimental

### 

#### Crystal data

C_10_H_8_N_2_·C_8_H_8_O_3_
*M* *_r_* = 308.33
Monoclinic, 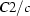

*a* = 25.3578 (6) Å
*b* = 10.2305 (2) Å
*c* = 14.2546 (4) Å
β = 122.321 (2)°
*V* = 3125.03 (15) Å^3^
*Z* = 8
Mo *K*α radiation
μ = 0.09 mm^−1^
*T* = 296 K
0.25 × 0.14 × 0.06 mm

#### Data collection

Bruker APEXII CCD area-detector diffractometer
Absorption correction: multi-scan (*SADABS*; Sheldrick, 1996 ▶) *T*_min_ = 0.984, *T*_max_ = 0.994
24334 measured reflections
3650 independent reflections
2128 reflections with *I* > 2σ(*I*)
*R*_int_ = 0.043

#### Refinement

*R*[*F* ^2^ > 2σ(*F* ^2^)] = 0.047
*wR*(*F* ^2^) = 0.139
*S* = 1.06
3650 reflections
214 parameters
H atoms treated by a mixture of independent and constrained refinement
Δρ_max_ = 0.15 e Å^−3^
Δρ_min_ = −0.16 e Å^−3^

### 

Data collection: *APEX2* (Bruker, 2006 ▶); cell refinement: *SAINT* (Bruker, 2006 ▶); data reduction: *SAINT*; program(s) used to solve structure: *SHELXS97* (Sheldrick, 2008 ▶); program(s) used to refine structure: *SHELXL97* (Sheldrick, 2008 ▶); molecular graphics: *SHELXTL* (Sheldrick, 2008 ▶); software used to prepare material for publication: *SHELXL97*.

## Supplementary Material

Crystal structure: contains datablocks I, global. DOI: 10.1107/S1600536810018842/pv2281sup1.cif

Structure factors: contains datablocks I. DOI: 10.1107/S1600536810018842/pv2281Isup2.hkl

Additional supplementary materials:  crystallographic information; 3D view; checkCIF report

## Figures and Tables

**Table 1:** Hydrogen-bond geometry (Å, °)

*D*—H⋯*A*	*D*—H	H⋯*A*	*D*⋯*A*	*D*—H⋯*A*
O2—H2*O*⋯N2^i^	0.97 (2)	1.71 (2)	2.679 (2)	179 (2)
O3—H3*O*⋯N1^ii^	0.87 (2)	1.86 (2)	2.728 (2)	171 (2)

Symmetry codes: (i) 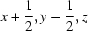; (ii) 
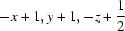
.

**Fig. 1.**
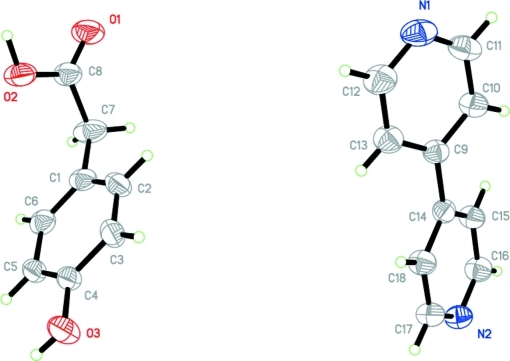
The molecular structure of the title complex. Displacement ellipsoids are drawn at the 30% probability level.

**Fig. 2.**
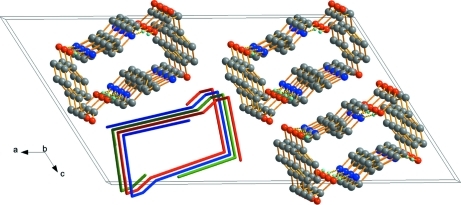
The packing plot of the complex showing triple-helix chains.

## supplementary crystallographic information

### Comment

The design and synthesis of coordination complexes have absorbed considerable attention due to their diverse structures with 4,4'-bipyridine linker (Han *et al.*, 2009). Moreover, hydrogen bond can also play a role in forming motif structures (Tomura & Yamashita, 2001). We have designed the title complex in an attempt to prepare crystaline magnetic materials and report its crystal structure in this paper.

The structure of the title complex is shown in Fig. 1, which reveals that it contains 2-(4-hydroxyphenyl)acetic acid and 4,4'-bipyridine in a ratio of 1:1. In the molecule of 2-(4-hydroxyphenyl)acetic acid, the acetyl torsion angle C(1)—C(7)—C(8)—O(2) is -32.1 (3) °, and in the molecule of 4,4'-bipyridine, the dihedral angle between the two pyridine rings is 23.41 (10) °. The two molecules arranged in the crystal at regular intervals with two O—H···N hydrogen bonds. The end to end hydrogen-bonding interactions lead to the formation of a one-dimensional triple-helix structure framework along the *b*-axis, Fig 2. Between adjacent triple-helix chains there exist weak π—π interactions.

### Experimental

2-(4-hydroxyphenyl)acetic acid (0.152 g, 1 mmol) and 4,4'-bipyridine (0.156 g, 1 mmol) were added to a mixed solution of ethanol (20 ml) and water (10 ml) with Cu(SO_4_)_2_ (0.127 g, 0.5 mmol) under stirred conditions at room temperature. A few minutes later a lot of blue deposit appeared. After the deposit was filtered out, a light blue solution was kept for evaporating. Colorless single crystals of the title complex were obtained about 19 days later.

### Refinement

The H atoms bonded to C atoms were positioned geometrically and refined using a riding model with C—H = 0.93 and 0.97 Å for methylene and aryl H-atoms, respectively and *U*_iso_(H) = 1.2*U*_eq_(C). The H atoms bonded to O atoms were located in a difference Fourier map and refined without restraints.

### Crystal data

**Table:**

C_10_H_8_N_2_·C_8_H_8_O_3_	*F*(000) = 1296
*M**_r_* = 308.33	*D*_x_ = 1.311 Mg m^−^^3^
Monoclinic, *C*2/*c*	Mo *K*α radiation, λ = 0.71073 Å
Hall symbol: -C 2yc	Cell parameters from 3274 reflections
*a* = 25.3578 (6) Å	θ = 1.9–27.7°
*b* = 10.2305 (2) Å	µ = 0.09 mm^−^^1^
*c* = 14.2546 (4) Å	*T* = 296 K
β = 122.321 (2)°	Block, colourless
*V* = 3125.03 (15) Å^3^	0.25 × 0.14 × 0.06 mm
*Z* = 8	

### Data collection

**Table:**

Bruker APEXII CCD area-detector diffractometer	3650 independent reflections
Radiation source: fine-focus sealed tube	2128 reflections with *I* > 2σ(*I*)
graphite	*R*_int_ = 0.043
φ & ω scans	θ_max_ = 27.7°, θ_min_ = 1.9°
Absorption correction: multi-scan (*SADABS*; Sheldrick, 1996)	*h* = −32→32
*T*_min_ = 0.984, *T*_max_ = 0.994	*k* = −13→13
24334 measured reflections	*l* = −18→18

### Refinement

**Table:**

Refinement on *F*^2^	Primary atom site location: structure-invariant direct methods
Least-squares matrix: full	Secondary atom site location: difference Fourier map
*R*[*F*^2^ > 2σ(*F*^2^)] = 0.047	Hydrogen site location: inferred from neighbouring sites
*wR*(*F*^2^) = 0.139	H atoms treated by a mixture of independent and constrained refinement
*S* = 1.06	*w* = 1/[σ^2^(*F*_o_^2^) + (0.065*P*)^2^ + 0.2994*P*] where *P* = (*F*_o_^2^ + 2*F*_c_^2^)/3
3650 reflections	(Δ/σ)_max_ < 0.001
214 parameters	Δρ_max_ = 0.15 e Å^−^^3^
0 restraints	Δρ_min_ = −0.16 e Å^−^^3^

### Special details

**Table:**

Geometry. All esds (except the esd in the dihedral angle between two l.s. planes) are estimated using the full covariance matrix. The cell esds are taken into account individually in the estimation of esds in distances, angles and torsion angles; correlations between esds in cell parameters are only used when they are defined by crystal symmetry. An approximate (isotropic) treatment of cell esds is used for estimating esds involving l.s. planes.
Refinement. Refinement of *F*^2^ against ALL reflections. The weighted *R*-factor wR and goodness of fit *S* are based on *F*^2^, conventional *R*-factors *R* are based on *F*, with *F* set to zero for negative *F*^2^. The threshold expression of *F*^2^ > σ(*F*^2^) is used only for calculating *R*-factors(gt) etc. and is not relevant to the choice of reflections for refinement. *R*-factors based on *F*^2^ are statistically about twice as large as those based on *F*, and *R*- factors based on ALL data will be even larger.

### Fractional atomic coordinates and isotropic or equivalent isotropic displacement parameters (Å^2^)

**Table:**

	*x*	*y*	*z*	*U*_iso_*/*U*_eq_	
C1	0.54789 (7)	0.32235 (16)	0.04851 (15)	0.0637 (5)	
C2	0.54036 (7)	0.28856 (15)	0.13430 (17)	0.0674 (5)	
H2A	0.5211	0.2098	0.1308	0.081*	
C3	0.56064 (8)	0.36856 (15)	0.22505 (16)	0.0624 (5)	
H3A	0.5554	0.3431	0.2822	0.075*	
C4	0.58890 (7)	0.48680 (14)	0.23132 (14)	0.0540 (4)	
C5	0.59659 (7)	0.52233 (14)	0.14611 (14)	0.0555 (4)	
H5A	0.6156	0.6014	0.1494	0.067*	
C6	0.57611 (8)	0.44072 (16)	0.05596 (15)	0.0618 (4)	
H6A	0.5814	0.4660	−0.0011	0.074*	
C7	0.52498 (9)	0.23409 (19)	−0.05088 (17)	0.0888 (7)	
H7A	0.4826	0.2074	−0.0765	0.107*	
H7B	0.5235	0.2843	−0.1100	0.107*	
C8	0.56331 (9)	0.11343 (17)	−0.03167 (16)	0.0686 (5)	
C9	0.27818 (7)	0.03470 (14)	0.13722 (13)	0.0531 (4)	
C10	0.24522 (9)	−0.08224 (16)	0.10478 (15)	0.0668 (5)	
H10A	0.2019	−0.0821	0.0680	0.080*	
C11	0.27717 (11)	−0.19771 (17)	0.12756 (17)	0.0777 (6)	
H11A	0.2543	−0.2749	0.1067	0.093*	
C12	0.37044 (10)	−0.09378 (19)	0.20793 (17)	0.0780 (6)	
H12A	0.4136	−0.0968	0.2423	0.094*	
C13	0.34242 (8)	0.02609 (17)	0.19086 (15)	0.0669 (5)	
H13A	0.3666	0.1015	0.2153	0.080*	
C14	0.24634 (7)	0.16282 (14)	0.11616 (13)	0.0510 (4)	
C15	0.18450 (8)	0.17940 (15)	0.03144 (14)	0.0600 (4)	
H15A	0.1621	0.1097	−0.0147	0.072*	
C16	0.15638 (8)	0.29964 (16)	0.01587 (15)	0.0656 (5)	
H16A	0.1144	0.3078	−0.0394	0.079*	
C17	0.24598 (9)	0.38940 (16)	0.15596 (15)	0.0657 (5)	
H17A	0.2678	0.4619	0.1983	0.079*	
C18	0.27730 (8)	0.27190 (15)	0.17954 (14)	0.0616 (4)	
H18A	0.3188	0.2660	0.2375	0.074*	
N1	0.33881 (9)	−0.20646 (15)	0.17762 (14)	0.0809 (5)	
N2	0.18645 (7)	0.40479 (13)	0.07623 (12)	0.0636 (4)	
O1	0.54120 (7)	0.01483 (14)	−0.08188 (14)	0.1052 (6)	
O2	0.62304 (6)	0.12757 (12)	0.04163 (12)	0.0847 (5)	
H2O	0.6456 (10)	0.046 (2)	0.0532 (17)	0.102*	
O3	0.60808 (6)	0.56199 (12)	0.32305 (11)	0.0733 (4)	
H3O	0.6234 (10)	0.635 (2)	0.3150 (17)	0.088*	

### Atomic displacement parameters (Å^2^)

**Table:**

	*U*^11^	*U*^22^	*U*^33^	*U*^12^	*U*^13^	*U*^23^
C1	0.0446 (9)	0.0533 (9)	0.0674 (12)	0.0112 (7)	0.0128 (8)	−0.0090 (8)
C2	0.0489 (10)	0.0440 (9)	0.0873 (13)	−0.0055 (7)	0.0218 (9)	−0.0050 (9)
C3	0.0525 (10)	0.0557 (9)	0.0743 (12)	−0.0072 (7)	0.0308 (9)	0.0034 (8)
C4	0.0497 (9)	0.0439 (8)	0.0632 (11)	−0.0009 (6)	0.0266 (8)	−0.0024 (7)
C5	0.0510 (9)	0.0436 (8)	0.0665 (11)	−0.0021 (6)	0.0279 (8)	0.0006 (7)
C6	0.0531 (10)	0.0616 (10)	0.0626 (11)	0.0104 (7)	0.0255 (9)	0.0015 (8)
C7	0.0684 (13)	0.0733 (12)	0.0792 (13)	0.0164 (10)	0.0090 (10)	−0.0249 (10)
C8	0.0630 (12)	0.0584 (10)	0.0692 (12)	0.0031 (8)	0.0252 (10)	−0.0127 (9)
C9	0.0596 (10)	0.0485 (8)	0.0501 (9)	0.0060 (7)	0.0286 (8)	0.0056 (7)
C10	0.0709 (12)	0.0541 (10)	0.0711 (12)	0.0042 (8)	0.0350 (10)	0.0022 (8)
C11	0.0991 (16)	0.0501 (10)	0.0807 (13)	0.0072 (10)	0.0461 (12)	0.0066 (9)
C12	0.0722 (13)	0.0720 (13)	0.0792 (13)	0.0231 (10)	0.0333 (11)	0.0192 (10)
C13	0.0621 (11)	0.0595 (10)	0.0687 (12)	0.0107 (8)	0.0280 (10)	0.0110 (8)
C14	0.0547 (9)	0.0484 (8)	0.0533 (9)	0.0038 (7)	0.0311 (8)	0.0066 (7)
C15	0.0599 (10)	0.0501 (9)	0.0636 (11)	0.0012 (7)	0.0288 (9)	−0.0005 (8)
C16	0.0555 (10)	0.0618 (10)	0.0669 (11)	0.0091 (8)	0.0243 (9)	0.0035 (9)
C17	0.0690 (12)	0.0540 (9)	0.0680 (11)	0.0023 (8)	0.0325 (10)	−0.0062 (8)
C18	0.0562 (10)	0.0567 (9)	0.0624 (11)	0.0022 (7)	0.0253 (9)	−0.0027 (8)
N1	0.1009 (14)	0.0620 (10)	0.0767 (11)	0.0285 (9)	0.0454 (10)	0.0179 (8)
N2	0.0656 (10)	0.0546 (8)	0.0665 (9)	0.0094 (7)	0.0327 (8)	−0.0004 (7)
O1	0.0818 (10)	0.0680 (9)	0.1354 (14)	−0.0077 (7)	0.0376 (9)	−0.0416 (9)
O2	0.0640 (9)	0.0596 (7)	0.0937 (10)	0.0124 (6)	0.0175 (8)	−0.0179 (7)
O3	0.0921 (10)	0.0576 (7)	0.0738 (8)	−0.0186 (6)	0.0467 (8)	−0.0124 (6)

### Geometric parameters (Å, °)

**Table:**

C1—C2	1.379 (3)	C10—C11	1.370 (2)
C1—C6	1.382 (2)	C10—H10A	0.9300
C1—C7	1.510 (2)	C11—N1	1.330 (2)
C2—C3	1.377 (2)	C11—H11A	0.9300
C2—H2A	0.9300	C12—N1	1.337 (3)
C3—C4	1.384 (2)	C12—C13	1.372 (2)
C3—H3A	0.9300	C12—H12A	0.9300
C4—O3	1.3631 (19)	C13—H13A	0.9300
C4—C5	1.378 (2)	C14—C18	1.385 (2)
C5—C6	1.380 (2)	C14—C15	1.386 (2)
C5—H5A	0.9300	C15—C16	1.379 (2)
C6—H6A	0.9300	C15—H15A	0.9300
C7—C8	1.503 (2)	C16—N2	1.333 (2)
C7—H7A	0.9700	C16—H16A	0.9300
C7—H7B	0.9700	C17—N2	1.327 (2)
C8—O1	1.190 (2)	C17—C18	1.380 (2)
C8—O2	1.309 (2)	C17—H17A	0.9300
C9—C13	1.384 (2)	C18—H18A	0.9300
C9—C10	1.389 (2)	O2—H2O	0.97 (2)
C9—C14	1.484 (2)	O3—H3O	0.87 (2)
			
C2—C1—C6	117.60 (16)	C11—C10—H10A	120.3
C2—C1—C7	121.07 (18)	C9—C10—H10A	120.3
C6—C1—C7	121.33 (19)	N1—C11—C10	124.19 (18)
C3—C2—C1	121.66 (16)	N1—C11—H11A	117.9
C3—C2—H2A	119.2	C10—C11—H11A	117.9
C1—C2—H2A	119.2	N1—C12—C13	123.29 (19)
C2—C3—C4	120.03 (17)	N1—C12—H12A	118.4
C2—C3—H3A	120.0	C13—C12—H12A	118.4
C4—C3—H3A	120.0	C12—C13—C9	119.99 (17)
O3—C4—C5	123.41 (14)	C12—C13—H13A	120.0
O3—C4—C3	117.52 (16)	C9—C13—H13A	120.0
C5—C4—C3	119.07 (15)	C18—C14—C15	116.89 (14)
C4—C5—C6	120.07 (14)	C18—C14—C9	121.49 (14)
C4—C5—H5A	120.0	C15—C14—C9	121.61 (14)
C6—C5—H5A	120.0	C16—C15—C14	119.74 (16)
C5—C6—C1	121.56 (17)	C16—C15—H15A	120.1
C5—C6—H6A	119.2	C14—C15—H15A	120.1
C1—C6—H6A	119.2	N2—C16—C15	123.21 (16)
C8—C7—C1	115.68 (15)	N2—C16—H16A	118.4
C8—C7—H7A	108.4	C15—C16—H16A	118.4
C1—C7—H7A	108.4	N2—C17—C18	123.72 (16)
C8—C7—H7B	108.4	N2—C17—H17A	118.1
C1—C7—H7B	108.4	C18—C17—H17A	118.1
H7A—C7—H7B	107.4	C17—C18—C14	119.42 (16)
O1—C8—O2	123.08 (17)	C17—C18—H18A	120.3
O1—C8—C7	122.41 (17)	C14—C18—H18A	120.3
O2—C8—C7	114.47 (15)	C11—N1—C12	116.44 (15)
C13—C9—C10	116.75 (15)	C17—N2—C16	116.96 (14)
C13—C9—C14	121.38 (14)	C8—O2—H2O	111.5 (12)
C10—C9—C14	121.87 (15)	C4—O3—H3O	108.1 (14)
C11—C10—C9	119.30 (18)		
			
C6—C1—C2—C3	−0.7 (2)	N1—C12—C13—C9	−1.8 (3)
C7—C1—C2—C3	−179.55 (15)	C10—C9—C13—C12	1.1 (3)
C1—C2—C3—C4	0.6 (2)	C14—C9—C13—C12	−179.22 (16)
C2—C3—C4—O3	−179.97 (15)	C13—C9—C14—C18	−23.0 (2)
C2—C3—C4—C5	−0.3 (2)	C10—C9—C14—C18	156.71 (16)
O3—C4—C5—C6	179.78 (14)	C13—C9—C14—C15	156.28 (16)
C3—C4—C5—C6	0.2 (2)	C10—C9—C14—C15	−24.1 (2)
C4—C5—C6—C1	−0.2 (2)	C18—C14—C15—C16	−1.8 (2)
C2—C1—C6—C5	0.5 (2)	C9—C14—C15—C16	178.93 (15)
C7—C1—C6—C5	179.37 (15)	C14—C15—C16—N2	2.6 (3)
C2—C1—C7—C8	−74.3 (2)	N2—C17—C18—C14	1.4 (3)
C6—C1—C7—C8	106.8 (2)	C15—C14—C18—C17	−0.1 (2)
C1—C7—C8—O1	150.2 (2)	C9—C14—C18—C17	179.18 (15)
C1—C7—C8—O2	−32.1 (3)	C10—C11—N1—C12	0.9 (3)
C13—C9—C10—C11	0.4 (3)	C13—C12—N1—C11	0.8 (3)
C14—C9—C10—C11	−179.26 (16)	C18—C17—N2—C16	−0.7 (3)
C9—C10—C11—N1	−1.5 (3)	C15—C16—N2—C17	−1.3 (3)

### Hydrogen-bond geometry (Å, °)

**Table:**

*D*—H···*A*	*D*—H	H···*A*	*D*···*A*	*D*—H···*A*
O2—H2O···N2^i^	0.97 (2)	1.71 (2)	2.679 (2)	179 (2)
O3—H3O···N1^ii^	0.87 (2)	1.86 (2)	2.728 (2)	171 (2)

Symmetry codes: (i) *x*+1/2, *y*−1/2, *z*; (ii) −*x*+1, *y*+1, −*z*+1/2.
